# An improved ViT model for music genre classification based on mel spectrogram

**DOI:** 10.1371/journal.pone.0319027

**Published:** 2025-03-13

**Authors:** Pingping Wu, Weijie Gao, Yitao Chen, Fangfang Xu, Yanzhe Ji, Juan Tu, Han Lin

**Affiliations:** 1 Jiangsu Key Laboratory of Public Project Audit, School of Engineering Audit, Nanjing Audit University, Nanjing, China; 2 School of Computer Science, Nanjing Audit University, Nanjing, China; 3 School of Mechanical, Electrical and Information Engineering, Shandong University, Weihai, China; 4 Department of Acoustics, School of Physics, Nanjing University, Nanjing, China; Tongji University, CHINA

## Abstract

Automating the task of music genre classification offers opportunities to enhance user experiences, streamline music management processes, and unlock insights into the rich and diverse world of music. In this paper, an improved ViT model is proposed to extract more comprehensive music genre features from Mel spectrograms by leveraging the strengths of both convolutional neural networks and Transformers. Also, the paper incorporates a channel attention mechanism by amplifying differences between channels within the Mel spectrograms of individual music genres, thereby facilitating more precise classification. Experimental results on the GTZAN dataset show that the proposed model achieves an accuracy of 86.8%, paving the way for more accurate and efficient music genre classification methods compared to earlier approaches.

## 1. Introduction

Music serves as a powerful tool for alleviating stress and enhancing overall well-being, playing an indispensable role in promoting mental and emotional health. It offers individuals a therapeutic outlet to unwind, find solace, and cultivate a sense of inner peace amidst life’s challenges [1]. Effective music genre classification represents a critical area of research within the field of Music Information Retrieval (MIR), offering tremendous opportunities to enhance music discovery and support academic research. By developing robust classification algorithms and leveraging computational techniques, researchers and practitioners can unlock the transformative potential of MIR to enrich the lives of music creators and listeners alike [2].

Currently, music genre classification faces several key challenges. Firstly, relying on manual classification often leads to inefficiency, especially when dealing with tremendous volumes of music data, and results in significant subjective differences. While objective judgment standards could mitigate some issues, the inherent ambiguity in distinguishing similar music genres and the reliance on subjective pre-classification remain persistent challenges. Secondly, deep learning models struggle to achieve satisfactory classification results when trained on small datasets. In fact, they may even underperform compared to traditional machine learning classification methods [3]. Given the limited number of songs users interact with daily, achieving accurate music classification with small sample datasets becomes crucial in the realm of music information retrieval [4]. Lastly, many convolutional models used in music genre classification struggle to capture essential global feature information within music feature maps due to the limitations of convolutional computation. This limitation impedes their ability to effectively discern and classify music genres based on global characteristics. Addressing these challenges necessitates innovative approaches, including the development of more objective and standardized classification criteria, the design of specialized algorithms capable of learning from limited data, and the exploration of alternative computational architectures that can better capture global features within music compositions [5]. By tackling these issues, researchers can significantly advance the field of music genre classification and enhance the accuracy and efficiency of music information retrieval systems.

Indeed, the reliance on manual classification for music genre classification remains a prevalent practice in the field. However, automated classification methods have emerged as a promising solution, driving the advancement of music information retrieval (MIR) systems. In this field, the selection of appropriate audio features is crucial for improving classification accuracy. Using spectrograms as features proves effective in deep learning-based music genre classification tasks, as spectrograms accurately reflect audio-acoustic metrics’ characteristics. Due to the resemblance between audio spectrograms and RGB images, deep learning models commonly used in computer vision are suitable for music genre classification, particularly those based on Convolutional Neural Networks (CNNs) [6,7]. Many existing methods adapt CNN models originally designed for image recognition, modifying them to accommodate audio spectrograms, Mel spectrograms, and other visual features as input. Lee et al. proposed an automatic music genre classification method based on spectral analysis of modulation spectrum and Mel-frequency cepstral coefficients (MFCC) features [8]. Finding the musical features of hit songs would be of great benefit to the music industry, so Herremans et al. addressed this problem by focusing on the dance hit classification problem [9]. Choi et al. explored the automatic classification of audio signals into a hierarchical structure of music genres [10]. Nanni evaluated and compared many different acoustic and visual features of sound and fused these features and finally achieved 90.9% accuracy [11], Mousumi used Apache Spark to reduce the computation time of machine learning prediction and optimized the hyperparameters of random forests and finally obtained 90% accuracy on GTZAN dataset [12]. However, all of these models require extensive preprocessing of the audio data, involving multiple steps such as noise reduction, normalization, segmentation, feature extraction (e.g., MFCC, spectral contrast), dimensionality reduction, and feature selection.

In the surge of self-attention based mechanisms, the Vision Transformer (ViT) based model [13], is an alternative approach for learning visual representations by convolutional neural networks. Briefly, ViT divides an image into a series of non-overlapping chunks and then uses a multi-headed self-attentive representation in the Transformer module. The general direction is to increase the number of parameters in the ViT network to improve performance [14,15]. However, these performance improvements come at the cost of model size (network parameters) and latency. Yang proposed a method to improve music genre classification with convolutional neural networks [16]. Qiu used, more recently, the Transformer deep learning approach [17]. Because of its huge training parameters and the required arithmetic resources, the ViT model still has some application difficulties in the field of image processing [18]. In 2021, the Mobile-ViT model [19] was proposed by Apple, with its miniaturized and lightweight technical characteristics. The application of the Transformer in the field of image processing provides great convenience. However, the disadvantage of Vision Transformer is the lack of spatial induction bias information. But because of Vision Transformer’s advantage for overall feature extraction from images, ViT still has high heat in the vision field after it was proposed in 2020. ViT needs to learn more information from huge data, demand more arithmetic, and pre-train on huge datasets to achieve better results, which is difficult to achieve for small sample datasets [20].

To address the aforementioned challenges, this paper undertakes a comprehensive review of existing literature on music genre classification and proposes an improved ViT model built upon Mel spectrograms. The primary contributions of this study are delineated as follows: Firstly, this paper presents an improved ViT model that combines the strengths of convolutional neural networks and Transformers to improve feature extraction from Mel spectrograms, leading to a more robust representation of musical content for genre classification. Secondly, to further enhance classification accuracy, the improved ViT model is augmented with a multi-channel attention mechanism. This mechanism operates by selectively emphasizing inter-channel variations across the Mel spectrogram channels, thereby improving the model’s capacity to distinguish among music genres. Lastly, by applying this multi-channel attention mechanism, the model amplifies critical inter-channel disparities within the feature data. This selective amplification highlights essential spectral channels, thereby strengthening the model’s ability to classify music genres with higher discriminative power.

The remainder of the paper is structured as follows: Section 2 outlines the specifics of the proposed method. Section 3 details the experimental setup, presents the results obtained and offers analyses of these findings. Lastly, Section 4 presents the main conclusions drawn from the study and outlines potential avenues for future research.

## 2. Construction of the improved ViT model

**Fig 1:**
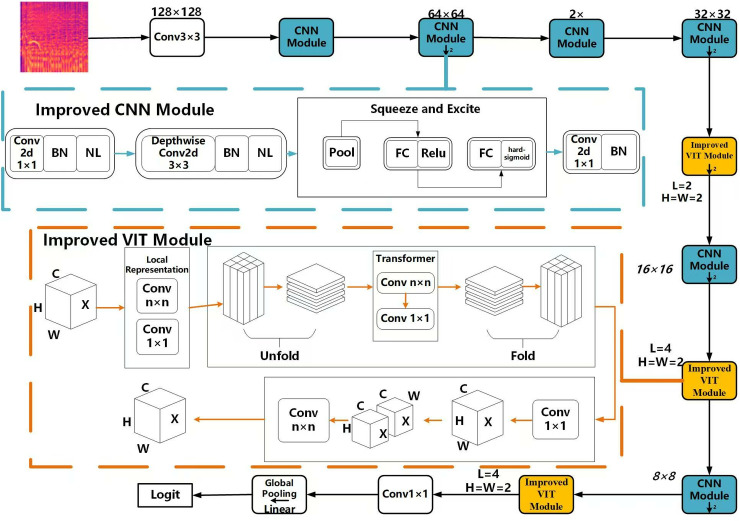
Diagram of the improved ViT model architecture. The whole framework mainly consists of two parts: the improved ViT module and the improved CNN module. In the improved ViT module part, the image data used is downsampled by convolution operation, and the data is reduced by using the unfold operation under the premise of ensuring the loss of less feature information. In the improved CNN module, the classification performance of the model is improved by introducing an augmented channel attention mechanism that focuses the attention on the differences between music genres in the Mel spectrogram. In the architecture diagram, L of the improved ViT module denotes the number of layers in each Transformer module, H is the length of the path size, and w is the width of the patch size. The down arrow in the module denotes the downsampling step with a step size of 2.

This paper introduces an improved ViT model that integrates a traditional convolutional neural network with the Transformer module. This integration capitalizes on the spatial induction bias features inherent in convolutional models and the attention mechanism’s ability to extract global feature characteristics effectively. Furthermore, an enhanced channel attention mechanism (ECAM) is introduced to optimize the performance of essential feature information within the channels of the Mel spectrogram. This enhancement aims to improve the ViT model’s computational efficiency and storage costs. The model architecture, depicted in Fig 1, primarily comprises the improved ViT module and the CNN module. The improved ViT module combines the strengths of Convolutional Neural Networks and the Transformer mechanism. Meanwhile, the CNN module incorporates an augmented channel attention mechanism, which focuses attention on the distinctions between musical genres in the Mel spectrograms, thereby further enhancing classification accuracy.

### 2.1. Improved ViT module

The standard Vision Transformer (ViT) model begins by dividing the input image into non-overlapping patches. Each patch is then flattened, and a linear projection is applied to obtain a token vector for each patch. These token vectors are subsequently concatenated to form a sequence of tokens. To retain positional information, a positional encoding is added to the token sequence. The resulting sequence is then processed through multiple layers of Transformer blocks, yielding the standard ViT representation [21].

In this paper, we propose an improved ViT module with a flowchart framework as shown in the orange dashed box in Fig 1, which mainly consists of three basic parts: local representations, transformer, and feature connection. In the Local representations part, the input image is divided into multiple local regions, and feature extraction is performed for each local region, then the features of all local regions are aggregated. In the Improved ViT module section, the image data used is downsampled by convolution operation to shrink the data and reduce the number of sampling points in the matrix, and a deconvolution operation is used to reduce the amount of data to be computed while ensuring that less feature information is lost. Finally, in the feature concatenation part, a 1×1 convolutional kernel is used to adjust the number of channels and reduce the data obtained in the second step into *C* channels, which is consistent with the feature maps of the data dimensional input in the first step. Then, the obtained feature maps are fused with the input feature maps by using the N×N convolutional layer (3×3which is used in this experiment).

In the modified ViT module, the input image is first divided into B×B blocks, each of which has a size of H×W. Then, each block is fed into an independent convolutional layer (Local Convolution), which performs convolutional operations only within the current block to obtain a local feature representation of the current block. In this way, the entire input image is converted into a B×B local feature map, where each local feature is associated with a corresponding region of the input image.

A schematic diagram of deconstruction in the improved ViT module is given in the improved ViT module section of Fig 1. Specifically, the deconstruction operation splits the input image into a plurality of chunks of size H×W and expands these chunks into column vectors, thereby forming a large matrix. Each column is a local block in the input image, and by processing these column vectors, the local features of the input image can be effectively extracted. For the data features already obtained by local modeling in the previous step, for each patch, the identically positioned tokens are spliced into a sequence and the improved structuring operation is implemented by a convolution operation. In terms of details, the deconvolution operation splices the identically positioned tokens into a sequence that reorganizes the output in the Transformer layer. The reconstruction method, on the other hand, collapses these sequences back into the original feature map. The main advantage of the Transformer layer in the improved ViT model, through global modeling, is to highlight the local features, by which the input image is divided into multiple chunks through local feature extraction, and then local feature extraction is performed on each chunk. Therefore, in the Transformer block, the query, key, and value of the multi-head self-attention mechanism are computed on the local features, which makes the improved ViT model better able to deal with the local features in the image and improves the accuracy of the model.

### 2.2. Improved CNN module in ViT

The CNN module primarily consists of three convolutional layers designed to sequentially extract and refine feature representations. The first layer is a 1×1 convolution with a ReLU6 activation function, which enhances the expressiveness of the feature space while controlling for data scaling. The second layer employs a convolution with ReLU6 activation and applies Batch Normalization to stabilize and accelerate training. This normalization minimizes internal covariate shifts, leading to faster convergence. Additionally, the input and output of the CNN module are residually connected to improve gradient flow and further expedite convergence. Finally, the third layer utilizes a convolution to downscale the feature map generated by the second layer, condensing the feature matrix for subsequent processing stages. In audio data, due to the similarity between Mel spectrograms, the use of the original CNN module does not have strong inter-channel important feature extraction capability. Therefore, an enhanced channel attention mechanism (ECAM) is proposed, which enhances and empowers the inter-channel important data features and expands the difference between important and unimportant information. Thus, the purpose of enhancing the classification effect is achieved. The augmented CNN module adopts a lightweight adaptive width convolution module, which can automatically adjust the number of channels in the convolution kernel to adapt to different levels of feature maps, thus improving the efficiency of the model. This module enhances relevant features and suppresses irrelevant ones by adaptively adjusting the weights of each channel. In the improved ViT model, the augmented channel mechanism is incorporated into the feedforward neural network to enhance the model’s ability to capture features at varying scales. Its specific structure is shown in the green dashed box in Fig 1.

(1) *Enhancement of channel attention mechanisms*

Enhanced Channel Attention Mechanism (ECAM) operates through three main stages: squeeze and excitation [22], followed by scale.

**Squeeze:** compresses the feature map into a vector by a global average pooling operation. That is, all the values on each channel of the feature map are averaged to get a vector of size C, where C is the number of channels of the feature map. The feature map is first compressed into a vector by global average pooling operation, denoted as:


$$z=\frac{1}{HW}\overset{H}{\underset{i=1}{\sum }}\overset{W}{\underset{j=1}{\sum }}X_{i,j}$$ (1)

Here $z\in R^{C}$represents the average value of each channel, and *H* represents the height of the input feature, and *W* represents the width of the input feature.

**Excitation:** this vector is learned through a two-layer fully connected network and a weight vector of size C is generated. This weight vector corresponds to the weight coefficients of each channel of the feature map and can be seen as an important weighting of the feature map channels. For the first fully connected layer, the number of nodes is equal to 1/4 of the number of feature matrix channels, and the number of nodes in the second fully connected layer is aligned with the number of feature matrix channels in the previous layer. This vector is learned through a two-layer fully connected network and a weight vector of size C is generated, denoted as:


$$s=\sigma \left(W_{2}\delta \left(W_{1}z\right)\right)$$ (2)

Here $W_{1}\in R^{c_{1}\times C}$ and $W_{2}\in R^{C\times c_{2}}$ are the weight matrices of the two fully connected layers, respectively, *δ* and *σ* denote the activation functions, which are usually chosen as ReLU and sigmoid functions. $c_{1}$ and $c_{2}$ are customized hyperparameters, usually set to values less than or equal to C. The output vector can be understood as the vector of features obtained through the learning of the two fully connected layers. The output vector can be interpreted as the weights about the importance of each channel number of the feature vector obtained through the learning of the two fully connected layers.

**Scale:** finally, the obtained weight vector is weighted with the original feature map to get the final feature map $Y\in R^{H\times W\times C}$, denoted as:


$$Y_{i}=\left(e^{s_{i}}-1\right)\times X_{i}$$ (3)

Here $X_{i}$ represents the value of the original feature map on the i-th channel, $Y_{i}$ denotes the data feature after weighting and $s_{i}$ denotes the weight coefficient of the corresponding channel, which are 0 and 1. The ECAM works by adaptively weighting the feature map channels to highlight the most important features for the given task. By using the Squeeze operation, we reduce the spatial complexity and focus on channel-wise information. The Excitation phase enables the model to learn which channels contribute more to the output, while the Scale operation emphasizes the most relevant channels, improving the model’s ability to focus on the most informative features. This mechanism enhances the model’s capacity to adaptively learn which aspects of the feature map are most important for classification. The ability to adjust the channel weights enables the model to automatically prioritize significant features and suppress less relevant ones, resulting in improved classification performance. Additionally, the use of the exponential function in the scaling step provides a strong, non-linear adjustment to the channel importance, ensuring that the model can effectively adapt to complex patterns in the data.

(2) *Enhanced CNN structure*

Compared with the original CNN module, the enhanced CNN module achieves the purpose of improving the accuracy by adding the SE [22] mechanism and updating the activation function, which is represented here by the nonlinear activation function (NL). While the early stages of the architecture, including the convolutional layer and depthwise separable convolutional layer, remain largely unchanged, significant modifications are introduced in the latter stages. Specifically, for the feature matrix obtained from the input data, an enhanced channel attention mechanism (ECAM) is applied. In the feature extraction process following downsampling, a weighted matrix is employed to reallocate the significance of data features, amplifying the contribution of critical information while suppressing less relevant features. Additionally, a squaring operation is introduced to accentuate the contrast between salient and redundant information. These refinements collectively enhance the model’s performance.

## 3. Experiments and analysis

### 3.1. Data sets and pre-processing

**Fig 2:**
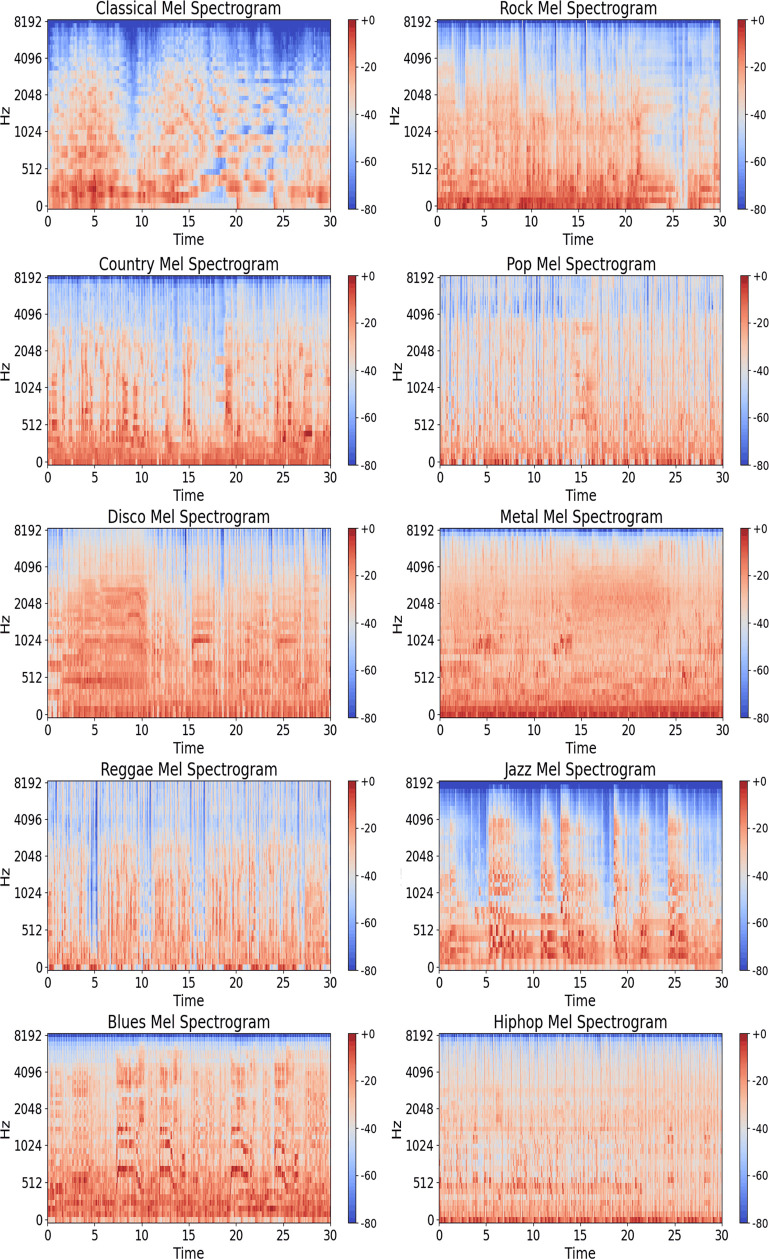
Examples of Mel spectrograms in the GTZAN dataset.

GTZAN dataset [24] is employed, which is a commonly used dataset in the field of music information retrieval. The GTZAN dataset contains 10 different types of music genres, including Blues, Classical, Country, Disco, Hip-hop, Metal, Jazz, Pop, Reggae, and Rock. Each genre contains 100 songs. Each song lasts 30 seconds, has a sampling rate of 22,050 Hz, and is stored in 16-bit AU file format. Since Mel spectrograms are an effective data feature for music genre classification in music classification experiments, this study uses Mel spectrograms obtained from the GTZAN dataset as the original data input and divides the dataset into 80% training set and 20% validation set. An example of the Mel spectrogram obtained from the GTZAN dataset is shown in Fig 2.

### 3.2. Ablation experiments

The environment settings and hardware configuration used for the specific experiments in this paper are shown in Table 1. In this section, the following experiments were conducted using the improved ViT depth model: (1) determining the optimal music genre classification chunk size by changing the size of the patches; (2) replacing the activation function in the CNN module to confirm the effect of the activation function on the accuracy of the Mel spectrograms; and (3) replacing a different CNN module to test the optimal network model based on the Mel spectrograms.

**Table 1:** The environment settings and hardware configuration.

Parameter	Specific Settings
Number of training epochs	100
Experimental environment	PyTorch 1.11.0, Python 3.8(ubuntu20.04), Cuda 11.3
GPU	RTX 3080 Ti(12GB) * 1
CPU	16 vCPU Intel(R) Xeon(R) Platinum 8350C CPU @ 2.60GHz
Memory size	64GB

In this paper, two metrics are used to quantitatively evaluate the performance of the improved ViT model for music genre classification: (1) the accuracy of the proportion of the total number of correct quantities predicted by the model, which is calculated by the following formula:


$$\begin{array}{c} Accuracy=\frac{\text{TP}+\text{TN}}{\text{TP}+\text{TN}+\text{FP}+\text{FN}} \end{array}$$ (4)

(2) Using training time per generation as an evaluation metric can be used to measure the time resources required to train the model.

After the following experiments, it is found that by replacing the CNN module of the model, there is indeed an improvement in the accuracy of the identification. With the introduction of the channel attention mechanism as well as the enhanced channel attention mechanism (ECAM), the classification accuracy was further improved by enhancing the weights of the important channels and suppressing the noise. Finally, by choosing the improved enhanced ViT model setting the patch size to 6, and using ReLu6 for the activation function, an accuracy of 86.8% was achieved.

#### 3.2.1. Patch size selection.

Finding the right Patch Size is important for the determination of the parameters of this model. Based on being able to save more feature information of the image, it does not waste the computational performance of the device, and at the same time, it can improve the accuracy rate. Thus, the main objective of this section is to find the best Patch size suitable for the Mel spectrogram based music genre classification based on the three models.

In this subsection, each module’s activation function is configured as ReLU6, and the classification performance of the models is compared by varying the Patch size parameter. For each of the four models, we evaluate model accuracy and average training time per epoch under different Patch sizes, as summarized in Table 2. The results indicate that, under consistent computational conditions, setting the Patch size to 4 yields the highest classification accuracy. This suggests that, in this experiment, using a Patch size of 4 preserves more of the original data’s feature information while efficiently balancing computational resource demands.

**Table 2:** Effect of different patch sizes on accuracy.

model	Patch size	Accuracy (%)	Training time (s)
Improved ViT	2	78.1	7.5
	4	**78.3**	5.5
	8	74.1	5.1
Improved ViT2	2	79.3	8.1
	4	**79.7**	6.2
	8	76.4	5.4
Improved ViT3	2	82.1	7.2
	4	83.4	5.3
	8	**85.1**	5.1
Improved ViT4	2	84.1	7.3
	4	**86.8**	5.7
	8	85.4	5.1

Improved ViT2, The CNN module lacks residual connections compared to Improved ViT; Improved ViT3, The CNN module incorporates a channel attention mechanism in addition to the features of Improved ViT2; Improved ViT4, The CNN module is enhanced with an augmented architecture, succeeding the modifications in Improved ViT3.

#### 3.2.2. Activation function selection.

In the experiments in this section, for the control variables, to ensure that the rest of the hardware and equipment conditions are the same, In the improved ViT network model, the effect of each activation function on the model is verified by comparing the effects of the different activation functions in the residual removal module and the augmentation of the CNN module, the specific experimental settings are set as follows, the Patch size is set to 4, and the number of training generations is 100. The results of the experiments are shown in Table 3, and the ReLu6 activation function is better than the other two activation functions for the internal comparison of all four models. Within the four models, the ReLu6 activation function outperforms the other two activation functions for the internal comparison of the models, and there is no big difference in the average time spent on training per generation. However, ReLu6 is indeed the best choice as an activation function for improving accuracy.

**Table 3:** Effect of different activation functions on accuracy.

Module name	Activation Function Setting	Accuracy (%)	Duration of each round (s)
Improved ViT	ReLu	76.6	5.8
	ReLu6	**78.3**	5.5
	Sigmoid	75.1	5.2
Improved ViT2	ReLu	78.2	5.1
	ReLu6	**79.7**	6.2
	Sigmoid	76.7	5.7
	ReLu	82.1	5.5
Improved ViT3	ReLu6	83.8	5.6
	Sigmoid	**85.1**	5.3
Improved ViT4	ReLu	84.1	5.5
	ReLu6	**86.8**	5.7
	Sigmoid	85.8	5.4

Improved ViT2, The CNN module lacks residual connections compared to Improved ViT; Improved ViT3, The CNN module incorporates a channel attention mechanism in addition to the features of Improved ViT2; Improved ViT4, The CNN module is enhanced with an augmented architecture, succeeding the modifications in Improved ViT3.

### 3.3. Comparison with other models

To evaluate the effectiveness and superiority of the proposed improved ViT4 model, we conducted experiments using some traditional machine learning methods, including Stochastic Gradient descent (SGD), K-Nearest Neighbors (KNN), Random Forest (RF), and Support Vector Machine (SVM). Secondly, comparisons were made with some Music neural network classification models of varying scales, including Music-Model-Small, Music-Model-Mid, Music-Model-Large, and Music-Model-XLarge. Finally, we evaluated the performance of popular state-of-the-art models, including Swin Transformer, Vision Transformer (ViT), and DeIT, in the music genre classification task. The experimental results are shown in Table 4.

**Table 4:** Performance of different methods.

Model	Accuracy (%)
SGD	65.5
KNN	69.5
RF	68.4
SVM	65.4
Music-Model-Small	72.1
Music-Model-Mid	74.6
Music-Model-Large	75.9
Music-Model-XLarge	76.1
Swin Transformer [23]	45.2
Vision Transformer (ViT) [13]	39.4
DeIT [14]	44.1
**Improved ViT4**	**86.8**

Abbreviations: SGD, Stochastic Gradient descent; KNN, K-Nearest Neighbors; RF, Random Forest; SVM, Support Vector Machine.

The experimental results show that the improved ViT4 model proposed in this paper achieves the best overall performance with the highest accuracy. The results show that the lack of global information in the convolutional network is compensated by combining the global feature advantage of the attention mechanism, which improves the problem of the large number of parameters of the attention mechanism. Combining the spatial induction bias features under the convolutional model with the global feature features extracted by the attention mechanism based on the improved ViT model, the accuracy is further improved by improving the CNN module and introducing the augmented channel mechanism, which leads to better performance. However, the proposed model has some limitations. First, its evaluation was limited to the GTZAN dataset, so further testing on more diverse datasets is needed. Second, the model’s increased complexity may result in higher computational costs, especially for large-scale or real-time applications. Additionally, while the hybrid CNN and attention mechanisms improve performance, the model may struggle with extreme noise or ambiguous genres.

## 4. Conclusions and future work

This paper presents an improved Vision Transformer (ViT) model with an integrated enhanced attention mechanism for music genre classification based on Mel spectrograms. First, we develop a hybrid model that combines ViT with convolutional neural networks (CNNs) to leverage the strengths of both architectures. ViT’s global perception ability captures overarching genre characteristics, while CNN’s local feature extraction enhances the model’s sensitivity to subtle musical details. Furthermore, an enhanced channel attention mechanism (ECAM) is incorporated into the hybrid ViT model. This mechanism amplifies channel feature weights by intensifying inter-channel differences in pooled data, thereby emphasizing shared characteristic features of the same music genre in the Mel spectrogram. Additionally, the improved ViT model is adaptive to the feature variations across different genres, improving classification accuracy. In future work, we plan to extend the scope of our research by incorporating additional publicly available music genre datasets to further evaluate the performance and generalizability of the proposed improved ViT4 model. We also aim to investigate the impact of various data augmentation techniques and domain adaptation strategies to enhance the model’s robustness in real-world scenarios.
